# An Exploratory Pilot Study to Investigate the Potential Relationship Between Porcine Reproductive and Respiratory Syndrome (PRRS) Virus Viremia Changes and Barn Manure Pit Management Procedures

**DOI:** 10.3390/pathogens15050453

**Published:** 2026-04-22

**Authors:** Claudio Marcello Melini, Mariana Kikuti, Xiaomei Yue, Cesar A. Corzo

**Affiliations:** Department of Veterinary Population Medicine, University of Minnesota, Saint Paul, MN 55108, USA; melin145@umn.edu (C.M.M.); mkikuti@umn.edu (M.K.); yue00106@umn.edu (X.Y.)

**Keywords:** PRRS, manure pumping, ammonia, hydrogen sulfide, viremia

## Abstract

Porcine reproductive and respiratory syndrome virus (PRRSV)-positive pigs can be exposed to high concentration of gases, such as ammonia and hydrogen sulfide when manure and urine stored in the pits beneath them is agitated and pumped. Such acute exposure can lead to adverse health effects such as respiratory system irritation. This study aimed to explore whether PRRS-positive growing pigs experience changes in viremia detection after manure pit agitation and pumping has been performed. To address this objective, two PRRS-positive growing pig farms were conveniently selected and visited twice during the week before and after manure agitation and pumping. Blood samples were collected to assess detection of viremia, using reverse transcription-polymerase chain reaction (RT-PCR). A logistic regression model was used to evaluate serum detection of PRRSV before and after the manure management event, accounting for pig age. Although PRRSV was detected in the serum of some pigs, under the conditions of the study, there were no statistically significant changes that would indicate that viremia detections change after the pigs had been exposed to barn manure pit agitation and pumping.

## 1. Introduction

Porcine reproductive and respiratory syndrome (PRRS) causative agent is an RNA virus belonging to the family *Arteriviridae* classified into two species: *Betaarterivirus europensis* (PRRSV-1) and *Betaarterivirus americense* (PRRSV-2) [1,2,3]. The primary replication targets of the virus are alveolar macrophages and cells of the monocyte and macrophage lineage [3,4]. PRRSV-2 viremia has been described to begin approximately six to 12 h after infection [3], with a typical duration of 14 to 30 days [5,6]. In pigs born to PRRSV- infected sows at 85 to 90 days of gestation, viremia can persist for up to 210 days post-infection [7]. Even when viremia is no longer detectable by molecular diagnostics, the virus can remain persistent in lymphoid tissues up to 250 days [3,4,6,8],

In the United States (U.S.), pig farms commonly store manure in pits located beneath barns or in external lagoons or storage ponds beside the barn [9]. In the Midwestern U.S., manure is typically used as cropland fertilizer during the fall, following harvest. A key management practice during this period is manure pit agitation, extraction, and land application, commonly referred to as “manure pumping” [9]. Notably, this process coincides with the annual PRRS epidemic, which generally begins between October and November. This period is characterized by declining environmental temperatures, conditions that may favor the environmental viability of the PRRS virus [10,11,12,13,14], thereby increasing the risk of transmission between farms [15].

During pit agitation and pumping, the levels of manure-produced gases, for instance ammonia (NH_3_) and hydrogen sulfide (H_2_S), can increase and can represent a hazard for both animals and humans [16]. Above-normal levels can cause adverse health effects, such as respiratory tract irritation and inflammation [17,18,19]. Such inflammation causes activation of macrophages [20] as type 1 helper T (Th1) cells recruit mononuclear cells/macrophages and lymphocytes and produce high levels of IFN-γ [21], which may ultimately provide more cells for the PRRS virus replication. A similar effect has been reported in poultry, when exposure to NH_3_ resulted in inflammation caused by an imbalance of regulatory T cells (Treg)/Th1 [22]. Despite these data, there is no information regarding the effects that these gases have on PRRS-positive pigs. Our hypothesis is whether PRRS-positive viremia dynamics are influenced by acute exposure to elevated levels of NH_3_ and H_2_S during manure pit agitation. Therefore, the objective of this exploratory field study was to evaluate if PRRSV detection in blood increases after manure pumping has occurred.

## 2. Materials and Methods

For this longitudinal exploratory pilot field study, two PRRSV-positive, single-sourced, conveniently selected growing pig farms located in Minnesota were enrolled. Pigs were sourced from PRRSV unstable sow farms as determined by the Morrison Swine Health Monitoring Project (MSHMP), a project that monitors their PRRS status weekly [23]. Weekly communication with enrolled growing pig sites allowed for farm visit scheduling around manure pumping practices between early October and mid-December. Each growing pig farm was visited four times during the study: twice before and twice after manure pumping. Visits occurred at approximately −7, −3, +3, and +7-days post manure pumping.

To monitor gas concentration levels, seven days before the first visit at each farm, two sets of sensors to record NH_3_ (Dräger PAC^®^ 8000, Dräger, Lübeck, Germany) and H_2_S (Dräger PAC^®^ 6500, Dräger, Lübeck, Germany) were installed in the estimated middle of the tunnel-ventilated barn room’s corridor, as this position would represent the entirety of the room’s airspace. Gas-measuring devices were placed inside a plastic bell cover to protect them from generated dust. They were hung at two distinct heights, 1.2 and 1.8 m, which are the approximate heights for both the finishing pig and human, respectively. Data were recorded every second from the sensor setup until the final sampling point. The NH_3_ sensor can detect the gas within the 0 and 300 parts per million (ppm) range, whereas the H_2_S sensor detection ranges between 0 and 100 ppm. At the end of the study, data were downloaded as a .csv file using a communication module (Dräger Communication [IR] Module for PAC^®^ Series Single Gas Monitors, Dräger, Lübeck, Germany) connected to a computer with transfer software (GasVision, Dräger Safety AG & Co. KGaA, Lübeck, Germany).

Each enrolled pig barn had two rooms divided by a solid wall, with one of them being selected for the study. The barn was stocked with pigs from the same sow source, and the age spread was between 7 and 14 days. Within each room, thirty conveniently selected pigs were ear-tagged for prospective monitoring throughout the course of the study. Pigs were in 20 pens across the whole room, with some pens having either 1 or 2 ear-tagged pigs. Blood samples were collected through venipuncture of the jugular vein using a 1 ½″ 20G or 18G needle (BD Vacutainer^®^ PrecisionGlide™, Becton, Dickinson and Company, Franklin Lakes, NJ, USA) attached to a vacutainer tube holder (BD Vacutainer^®^ one-use holder, Becton, Dickinson and Company, Franklin Lakes, NJ, USA), and a collection tube without anticoagulant (BD Vacutainer^®^ Serum Tubes, Becton, Dickinson and Company, Franklin Lakes, NJ, USA). Tubes were labeled according to the pig’s ear-tag, pen, farm, and collection day. All samples were stored and transported in a cooler with an estimated temperature of 4 °C to the food-centric corridor processing laboratory at the University of Minnesota (UMN) College of Veterinary Medicine.

Blood samples were centrifuged at 1500× *g* at 4 °C for 10 min. From each serum sample, two 500 µL aliquots were obtained, placed in microcentrifuge tubes (Research Grade Microcentrifuge 1.7 mL, DOT Scientific Inc., Burton, MI, USA), and frozen at −80 °C before the submission to the UMN Veterinary Diagnostic Laboratory (VDL) for PRRSV Reverse Transcription-PCR (RT-PCR). Briefly, high-throughput total nucleic acid extraction was performed using magnetic bead technology (MagMAX™ CORE Nucleic Acid Purification Kit, ThermoFisher Scientific Inc., Waltham, MA, USA), followed by RT-PCR using the VetMAX™ PRRSV EU & NA v3.0 kit (ThermoFisher Scientific Inc., Waltham, MA, USA). Interpretation of PRRSV RT-PCR Cycle threshold (Ct) values was done according to VDL criteria, where samples with a Ct equal or above 40 were considered negative whereas samples with a Ct below 40 were considered positive.

Statistical analysis was performed using a logistic regression, implemented with the glm function from the stats package in the R statistical software version 4.5.1 [24]. The Ct values from the PRRSV RT-PCR were used as the outcome and treated as a binary variable, as our goal was to determine whether viremia could be detected before and after manure pumping. Sampling visits were categorized into before and after manure pumping while adjusting for pig age. Gas concentration levels of NH_3_ and H_2_S were described in ppm for each sampling-visit day and for the day when manure pumping and agitation occurred.

## 3. Results

A total of 15 out of the 240 serum samples tested positive for PRRSV, with at least one viremic pig being detected in 5 out of the 8 visits. The number of positive pigs per visit ranged between 1 and 5, with Ct values ranging between 25.8 and 36.2. The number of PRRSV RT-PCR-positive samples decreased after manure pumping at both farms. Gas concentration data were not recorded by either visit 1 or 2 due to sensor malfunctioning (Table 1).

Due to unknown reasons, gas concentration data from Farm 1 sensors were not captured for both NH_3_ sensors during the first visit, and for the upper sensor during the second visit. For Farm 2, NH_3_ data were not captured for either sensor during the first and second visits, and data from both H_2_S sensors were missing during the first visit. The concentration levels ranged from 0.0 to 127.0 ppm for NH_3_, and from 0.0 to 23.1 ppm for H_2_S.

From the logistic regression model, neither the sampling visit (before or after manure pumping) (*p* = 0.26) nor pig age (*p* = 0.40) were significantly associated with the outcome.

## 4. Discussion

Our exploratory pilot study attempted to understand whether there was an association between exposure to gases released during manure pumping and an increase in the number of PRRSV-viremic pigs. While multiple years of data suggest that PRRSV occurrence in the fall may be related to specific industry practices such as manure pumping [15,25], the present study failed to find a relationship regarding a potential exacerbatory effect of manure pumping on the number of viremic pigs that could lead to more shedding and, thus, increased risk of regional viral dissemination. While it cannot be concluded why these two events were not found to be related, there are different potential explanations, including infectious status stage, mild gas concentration exposure, and sample size.

The two groups of pigs monitored in this study were sourced from PRRSV unstable herds; however, since these were weaned approximately at 4 weeks of age and the manure pumping event occurred 6 to 12 weeks later, it is possible that the proportion of viremic pigs had reached such a low level that the manure pumping event did not trigger an exacerbating effect. Furthermore, it is likely that some of the pigs in these two barns had already overcome the viremic phase and entered the persistence stage, which has been defined as infected pigs that no longer have viremia but continue to sequester the virus in lymphoid tissues [3,26]. Clearly, exposure to manure pumping gases of these non-viremic and potentially persistent pigs had no effect on viremia, such as a rebound effect represented by a biphasic viremia curve, which has been previously described [27,28,29]. The presence of rebound viremic pigs could lead to subpopulations posing a health management challenge, as the herd might be considered past the viremic phase but would still be infectious, representing a regional risk [28].

The study was designed with the underlying assumption that PRRSV-positive pigs would be acutely exposed to high levels of NH_3_ and H_2_S, leading to viremia rebound. During the initial sampling visits, some of the data were not recorded unbeknownst to the researchers, although concentrations were being measured and displayed in real time. Therefore, while the monitored pigs were exposed to manure pumping gases, the magnitude of such exposure was not clearly quantified. In addition, gas concentration levels can be affected by other simultaneous activities. One of these is ventilation management during manure pumping; for instance, at Farm 1, weather conditions favored the opening of curtains for longer periods, which ultimately improved air exchange in the barn. An increase in air exchange leads to better indoor air quality as gases can exit the building, which can explain why the NH_3_ and H_2_S concentrations during the event at this farm did not increase to expected levels. At Farm 2, the levels did increase to those expected during manure agitation and pumping [17], especially for NH_3_, but viremia detections were fewer after the event. At least two moments, concentration levels were detected to be above or close to 100 ppm, which can be attributed to the pumping event occurring during three consecutive days.

The lack of statistically significant findings regarding an increase in viremic pigs after manure pumping could be a result of the small sample size. The authors acknowledge that the inclusion of a larger number of farms and monitored pigs would have potentially yielded different results, as some groups of pigs at the time of manure pumping might have been in the early stages of their infectious period rather than the latter stages; however, limited resources only allowed for an exploratory study in these two conveniently selected farms.

This study has other limitations besides the sample size. One is related to the fact that the hypothesis is based on inflammatory exacerbation caused by manure pumping gases; however, our study did not collect immunological information, which would have been another method to confirm such an irritation effect and Th activation [30]. This is a factor that needs to be accounted for in future studies to improve not only the analysis but also to reach inference.

This leads researchers to believe that specialized equipment and a larger number of sensors are needed, as variations in readings can occur according to placement area [31]. However, it is important to mention that the exact threshold of gas concentration that would have a negative impact on health varies [32], especially as gas concentration during manure pumping, is not constant [31].

Nonetheless, the impact of manure pumping on pig health needs further understanding from a viral dynamic’s perspective, as this may affect persistence and transmission during this period of the year. Although our model yielded non-statistically significant results, these results should be interpreted with caution, as the mentioned limitations do not allow us to fully provide evidence that there is no effect of manure agitation and pumping on viremia detections.

## Figures and Tables

**Table 1:** Summary of porcine reproductive and respiratory syndrome virus RT-PCR-positive detection in serum, and gas concentration levels from each sensor, by farm and visit.

		Farm Number									
		1					2				
		Visit Number									
		1	2	Event	3	4	1	2	Event	3	4
Number of days between visit and manure pumping event		−8	−3	-	2	6	−7	−3	-	3	7
Age (in weeks)		10	11	11	11	12	16	16	17	17	18
PRRSV RT-PCR positive serum samples		1/30	5/30	-	3/30	0/30	0/30	4/30	-	2/30	0/30
PRRSV RT-PCR serum Ct values											
	Median	28.7	31.3	-	30.1	-	-	33.4	-	32.8	-
	Minimum	28.7	25.8	-	26.9	-	-	33.1	-	32.6	-
	Maximum	28.7	36.2		34.7	-	-	35.6		32.9	-
Ammonia (NH_3_) (ppm)											
Lower sensor (1.2 m)	Median	NR	4.2	0.0	0.0	0.0	NR	NR	7.0	0.0	0.0
	Minimum	NR	0.0	0.0	0.0	0.0	NR	NR	0.0.0	0.0	0.0
	Maximum	NR	17.1	18.3	4.9	8.8	NR	NR	127.0	18.6	5.1
Upper sensor (1.8 m)	Median	NR	NR	0.0	0.0	5.4	NR	NR	0.0	0.0	0.0
	Minimum	NR	NR	0.0	0.0	0.0	NR	NR	0.0	0.0	0.0
	Maximum	NR	NR	23.3	8.9	9.7	NR	NR	86.2	7.6	0.0
Hydrogen sulfide (H2_S_) (ppm)											
Lower sensor (1.2 m)	Median	0.0	0.5	0.0	0.0	0.4	NR	0.0	0.0	0.0	0.0
	Minimum	0.0	0.0	0.0	0.0	0.0	NR	0.0	0.0	0.0	0.0
	Maximum	0.0	1.4	1.4	0.4	0.7	NR	0.9	23.1	2.0	0.0
Upper sensor (1.8 m)	Median	0.0	0.0	0.0	0.0	0.0	NR	0.0	0.0	0.0	0.0
	Minimum	0.0	0.0	0.0	0.0	0.0	NR	0.0	0.0	0.0	0.0
	Maximum	0.0	1.4	1.5	0.0	0.5	NR	0.5	22.0	1.6	0.0

Abbreviation: Ct = Cycle threshold; NR = not recorded; ppm = parts per million; RT-PCR = reverse transcription polymerase chain reaction.

## Data Availability

The raw data supporting the conclusions of this article will be made available by the authors on request.

## Abbreviations

The following abbreviations are used in this manuscript:

PRRS	Porcine reproductive and respiratory syndrome
PRRSV	Porcine reproductive and respiratory syndrome virus
PCR	Polymerase chain reaction
RNA	Ribonucleic acid
US	United States
NH_3_	Ammonia
H_2_S	Hydrogen sulfide
MSHMP	Morrison swine health monitoring project
PPM	Parts per million
UMN	University of Minnesota
VDL	Veterinary diagnostic laboratory
RT-PCR	Reverse transcription polymerase chain reaction
CT	Cycle threshold
NR	Not recorded
